# How Did the COVID-19 Pandemic Affect Emergency Dental Trauma Settings in Permanent Dentition? A Retrospective Study

**DOI:** 10.3390/jcm13237066

**Published:** 2024-11-22

**Authors:** Florian Dudde, Manfred Giese, Oliver Schuck, Christina Krüger

**Affiliations:** 1 Department of Oral and Maxillofacial Surgery, Army Hospital Hamburg, 22049 Hamburg, Germany; 2 Department of Neurology, University Medical Center Hamburg-Eppendorf, 20246 Hamburg, Germany

**Keywords:** COVID-19, pandemic, dental trauma, patterns, distribution

## Abstract

**Background**: The purpose of this study was to examine how the COVID-19 pandemic influenced the patterns, distribution, and circumstances of dental trauma (DT) cases at a German cranio-maxillofacial trauma center. **Materials and Methods***:* This retrospective analysis compared DT cases from the PreCovid (PC) period (February 2019–January 2020) with those from the IntraCovid (IC) period (February 2020–January 2021). It included an examination of baseline characteristics, types of DT, circumstances leading to DT, and the treatment approaches applied. **Results**: In the IC period, there was an increase in the number of DT, a significant increase in uncomplicated crown fractures, crown–root fractures, subluxations, avulsions, alveolar fractures, combined tooth fractures and dislocations, and concomitant soft tissue injuries. There were no differences regarding the location of DT. During the IC period there was a significant reduction in sports accidents, road traffic accidents, interpersonal violence, and alcohol-related DT. Simultaneously, there was a marked increase in falls, syncopal episodes, home accidents, and DT incidents occurring on weekdays. Furthermore, during the IC period, the number of cases of DT increased in the morning and decreased at nighttime. **Conclusions**: The COVID-19 pandemic significantly affected the types of DT, the treatment approaches, and the circumstances under which DT occurred. Investigating these impacts can help to predict the effects of a future pandemic on DT and/or maxillofacial trauma and possibly reduce these effects through establishing appropriate preventive measures.

## 1. Introduction

Dental trauma (DT) is one of the most common causes of emergency presentation to a clinic with an oral and maxillofacial surgery department [1,2]. The causes of DT can be diverse [2,3]. The most common causes leading to DT include sports accidents, interpersonal violence, falls, and road traffic accidents by car, bike, and/or e-scooter [2,3,4]. Dental trauma can be differentiated into tooth fractures and tooth dislocations/periodontal trauma [5]. Furthermore, tooth fractures can be divided into uncomplicated crown fractures (enamel only or enamel + dentin), complicated crown fractures (enamel + dentin + pulp exposure), root fractures, as well as combined crown and root fractures [6,7]. The uncomplicated crown fracture represents the most common form of tooth fracture [8]. The different types of tooth fractures require different emergency therapies [7]. In the case of an uncomplicated crown fracture, a temporary cover of the dentin wound can be achieved by applying composite using an acid-etching technique [7,8,9]. Furthermore, the reattachment of broken tooth fragments with composite (after comprehensive disinfection and, for example, storage in a “DentoSafe-Box”) is also a therapeutic option [7,9,10].

For complicated crown fractures, prompt pulp capping is particularly essential in order to increase the probability of preserving the affected tooth (follow-up root canal treatment is often required) [9,10]. Tooth dislocations and/or periodontal trauma can be divided into tooth concussion, subluxation, intrusive/extrusive/lateral/palatal dislocation, and avulsion and alveolar fractures [7,11]. In addition to repositioning, splint therapy plays an essential role when treating periodontal trauma. Typically, semi-rigid/flexible splints (i.e., titanium trauma splint = TTS) are used, which are fixed to the teeth using composite via the common acid-etching technique to ensure that the periodontally traumatized tooth is splinted into an anatomically correct position [7,9,11,12]. However, the duration of TTS varies depending on the type and severity of periodontal trauma [13]. Alveolar fractures require rigid splinting and/or miniplate osteosynthesis using titanium plates and screws, depending on the extent of the fracture and accompanying injury [14].

The maxillary incisors are the most likely teeth to be affected by DT due to their slightly protruded position [15]. Accompanying injuries to the surrounding hard and soft tissue structures, such as the lip, jaw, and/or nose, are not uncommon [2].

The COVID-19 pandemic had an immense impact on the daily lives of many people, as well as on the global healthcare system [16,17]. The pandemic challenged the global health care system, facing challenges in the treatment of COVID-19-associated pneumonia and the maintenance of adequate intensive care capacity. Furthermore, the COVID-19 pandemic also had an immense impact on other medical specialties, such as maxillofacial surgery [18,19]. Nationwide lockdowns and social distancing led to a drastic reduction in leisure activities, road traffic movements, and community sports activities [20]. The impact of the COVID-19 pandemic has already been studied with regards to mandible fractures, midface fractures, and nose fractures [18,19,21]. However, it is currently unclear to what extent the COVID-19 pandemic affected emergency DT settings in maxillofacial surgery. Accordingly, this study aimed to examine the impact of the COVID-19 pandemic on DT distribution, the circumstances and patterns leading to DT, and the emergency treatment approaches used. It also sought to compare these findings with data from a cranio-maxillofacial trauma center from the PreCovid period.

## 2. Materials and Methods

### 2.1. Data Collection

This study reviewed patients who visited the Department of Oral and Maxillofacial Surgery with DT between February 2019 and January 2021. Patients were categorized into two cohorts based on their admission dates as follows: PreCovid (PC) (February 2019–January 2020) and IntraCovid (IC) (February 2020–January 2021). All participants were at least 18 years old and fully able to consent to the procedures and diagnostics. Exclusion criteria were incomplete documentation (patient records) and patients under 18 years of age. A total of 250 patients were included in this study.

### 2.2. Baseline Characteristics and Dental Trauma Patterns

Baseline characteristics, such as gender and age, were retrospectively gathered for each patient. Additionally, DT patterns were recorded for each patient as follows: type, trauma/tooth location, tooth dislocation, associated fractures, soft tissue injuries, and treatment type.

### 2.3. Circumstances of Dental Trauma

The circumstances leading to DT were evaluated for each patient, including factors such as falls, traffic accidents, sports injuries, interpersonal violence, and incidents at home.

### 2.4. Statistical Analysis

A descriptive analysis was conducted to present the patients’ baseline characteristics. Normally distributed continuous variables are shown as mean ± standard deviation, while binary variables are reported in absolute and relative frequencies. Comparisons of continuous variables were made using Student’s *t*-test, and binary variables were analyzed with the Chi-square test. A *p*-value of less than 0.05 was deemed statistically significant. All statistical analyses were performed using SPSS version 28.0 (IBM, Markham, ON, Canada).

## 3. Results

### 3.1. Baseline

A total of 250 patients were included in the present study. The mean age of the patients was 46.21 (±19.27) years (with a minimum age of 18 years, and a maximum of 92 years) (Table 1). The percentage of male patients was 64.8% (Table 1). The study population was divided into two cohorts (PreCovid, *n* = 122 vs. IntraCovid, *n* = 128) based on the patients’ date of admission.

### 3.2. Type of Dental Trauma

The mean number of affected teeth was 2.04 (±1.36) for all patients. In the IC period, patients tended to have higher numbers of traumatized teeth in comparison to the PC period (PC = 1.89 (±1.17) vs. IC = 2.19 (±1.52)) (Table 2). No significant differences were found regarding enamel fractures between the two cohorts (Table 2). However, uncomplicated crown fractures (enamel + dentin) showed significantly higher rates in the IC period (PC = 63.9% vs. IC = 76.6%, *p* = 0.021) (Table 2). In addition to that, in the IC period, patients presented with significantly higher frequencies of combined crown–root fractures of traumatized teeth (PC = 8.2% vs. IC = 43.4%, *p* ≤ 0.001) (Table 2). The rates of complicated crown fractures as well as root fractures were similar in both periods (Table 2).

Regarding the type of tooth dislocation, there were significant differences between the two cohorts. During the IC period, patients showed significantly higher rates of subluxated teeth (PC = 19.7% vs. IC = 32.8%, *p* = 0.018), tooth avulsion (PC = 13.1% vs. IC = 21.9%, *p* = 0.049), and fractures of the alveolar process (PC = 1.6% vs. IC = 12.5%, *p* ≤ 0.001) (Table 2). In addition to that, there were significantly higher frequencies of combined tooth fracture and tooth dislocation in the IC period (PC = 55.7% vs. IC = 67.2%, *p* = 0.047) (Table 2). Meanwhile, in the PC period, patients tended to present with significantly higher numbers of intrusive and palatal luxation of traumatized teeth (Table 2). No significant differences were found regarding the rates of tooth concussion and extrusive or lateral luxation (Table 2).

**Figure 1:**
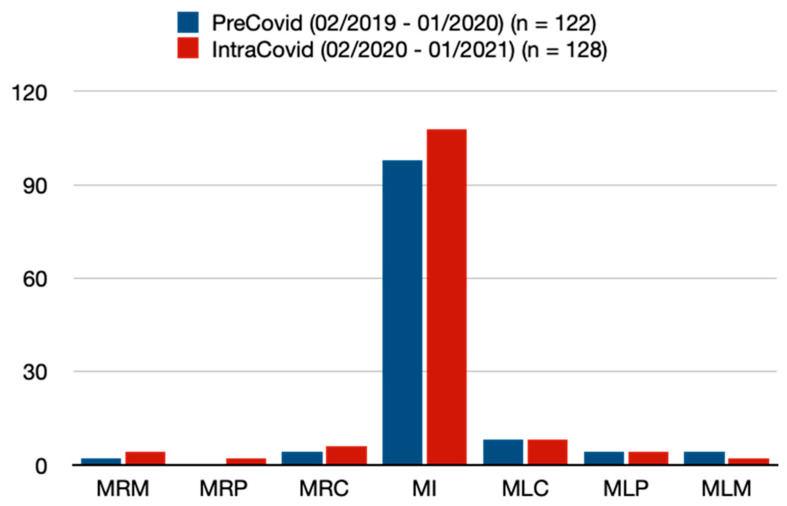
Traumatized maxillary teeth. MRM = maxillary right molar, MRP = maxillary right premolar, MRC = maxillary right caninus, MI = maxillary incisor, MLC = maxillary left caninus, MLP = maxillary left premolar, MLM = maxillary left molar. Note: Data are presented as absolute values/number of teeth.

**Figure 2:**
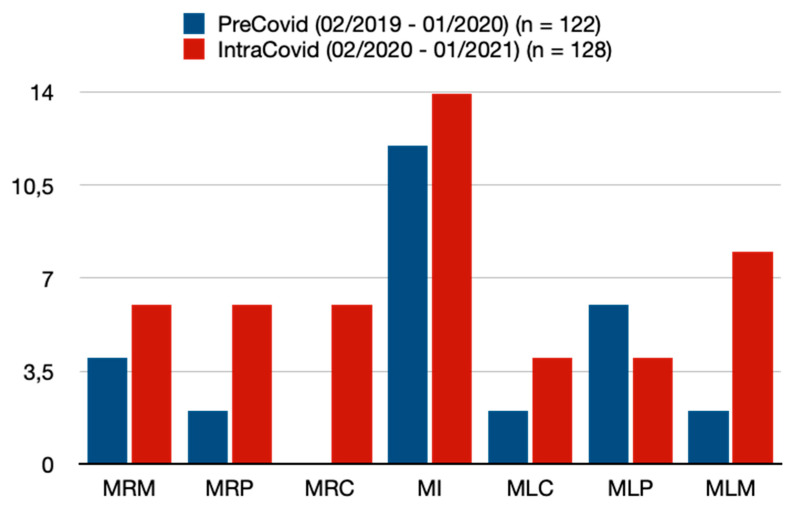
Traumatized mandibular teeth. MRM = mandibular right molar, MRP = mandibular right premolar, MRC = mandibular right caninus, MI = mandibular incisor, MLC = mandibular left caninus, MLP = mandibular left premolar, MLM = maxillary left molar. Note: Data are presented as absolute values/number of teeth.

Regarding the location/tooth number of traumatized teeth, there were similar frequencies between the two periods (Table 3, Figure 1 and Figure 2). The most common traumatized teeth were maxillary incisors (PC = 80.3% vs. IC = 84.4%, *p* = 0.401) (Table 3, Figure 1 and Figure 2). Mandibular incisors proved to be the second most common traumatized teeth (PC = 9.8% vs. IC = 10.9%) within this study (Table 3, Figure 1 and Figure 2). A total of 16 patients (6.4%) presented with a combination of traumatized maxillary and mandibular teeth (Table 3). In the IC period, patients tended to have higher rates of concomitant facial fractures (i.e., mandibular fracture); however, this was without statistical significance (Table 3). Furthermore, patients in the IC period showed significantly higher frequencies of concomitant soft tissue injury/laceration (PC = 55.7% vs. IC = 68.8%, *p* = 0.034) (Table 3).

### 3.3. Emergency Treatment of Dental Trauma

Regarding the emergency treatment of DT, there were significant differences between the two periods (Table 4). In the IC period, patients were more often treated with pulp capping (PC = 23.0% vs. IC = 43.8%, *p* = < 0.001) as well as with rigid splinting (i.e., concomitant alveolar fracture treatment) (PC = 3.3% vs. IC = 14.1%, *p* = 0.003) (Table 4). No significant differences were found between the two periods when using composite as an emergency treatment for fractured teeth or when using semirigid splints (i.e., TTS) for dislocated teeth after repositioning (Table 4). However, in the IC period, semirigid splinting was used more often (PC = 57.4% vs. IC = 62.5%, *p* = 0.079) (Table 4).

### 3.4. Circumstances of Dental Trauma

**Figure 3:**
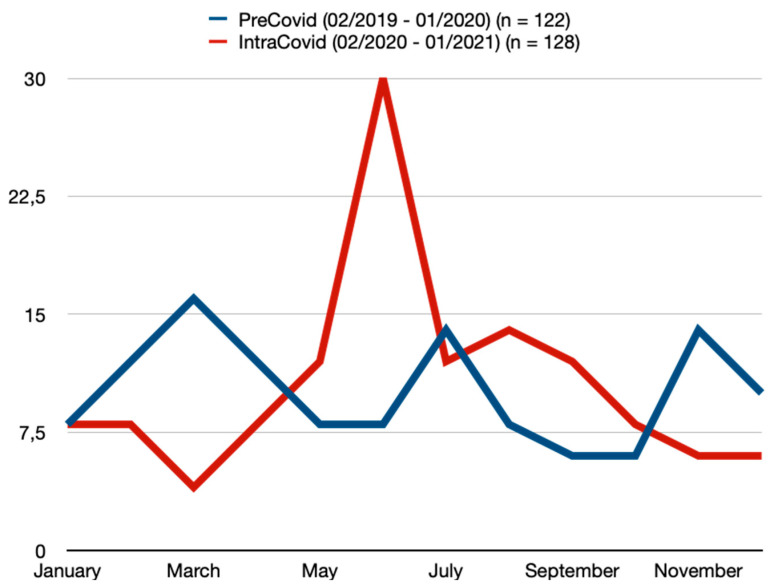
Monthly distribution of dental trauma.

Significant differences were found between the two periods regarding the circumstances leading to DT (Table 5). A significant increase was observed in falls (PC = 13.1% vs. IC = 46.9%, *p* ≤ 0.001), syncope (PC = 1.6% vs. IC = 17.2%, *p* ≤ 0.001), as well as accidents at home (PC = 20.5% vs. IC = 42.2%, *p* ≤ 0.001) leading to DT (Table 5). Meanwhile, a significant decrease was observed in sports accidents (PC = 39.3% vs. IC = 10.9%, *p* ≤ 0.001), road traffic accidents (PC = 39.3% vs. IC = 28.1%, *p* = 0.008), interpersonal violence (PC = 24.6% vs. IC = 9.4%, *p* = 0.001), and alcohol-related accidents (PC = 49.2% vs. IC = 9.4%, *p* ≤ 0.001) leading to DT (Table 5). Furthermore, the rates of domestic violence causing DT doubled within the IC period; however, this was without statistical significance (Table 5). No differences were found regarding the monthly distribution of DT (Table 5, Figure 3). However, June and July were the months with the highest number of reported DT cases (Table 5, Figure 3). During the IC period, there was a notable increase in DT occurrences on weekdays (PC = 52.5% vs. IC = 68.8%, *p* = 0.008) (Table 5). Additionally, a shift in the time of day for DT incidents was observed (Table 5). There was a significant rise in accidents during the morning hours (8 a.m.–4 p.m.) (PC = 11.5% vs. IC = 53.1%, *p* < 0.001), alongside a concurrent decrease in nighttime accidents (12 a.m.–8 a.m.) (PC = 37.7% vs. IC = 6.3%, *p* < 0.001) (Table 5).

## 4. Discussion

The objective of this study was to examine the effects of the COVID-19 pandemic on DT distribution, the circumstances and patterns associated with DT, and emergency treatment methods. Additionally, it aimed to compare these findings with data from the PreCovid period. In the present study, there was a subtle increase in the total number of patients with DT presenting as an emergency at the maxillofacial surgery department during the IC period. While comparable studies have shown decreases in the number of maxillofacial fractures, the prevalence of DT appears to remain stable during the IC period [18,19,21]. The results of the present study are also in line with the findings of a recent review by Campos et al. [22]. In their review, it was stated that the COVID-19 pandemic has neither impacted the frequency nor the type of DT compared with previous eras. However, with regards to the type of DT, the results of the present study contradict these findings [22]. In the present study, there was an increase in the number of traumatized teeth in the IC period (PC = 1.89 vs. IC = 2.19, *p* = 0.079). The present study also showed a significant increase in crown–root fractures and uncomplicated crown fractures in the pandemic era. The uncomplicated crown fracture (enamel + dentin) is considered the most common tooth fracture [8]. An increase in this type of tooth fracture was also demonstrated in a comparable study from the United Kingdom, but with a smaller population size [23]. Wooley et al. have also shown a large increase in the intrusive luxation of teeth associated with an increase in falls and higher axial trauma exposition to the teeth during the COVID-19 pandemic [23]. Even though the present study did not reveal an increase in intrusive luxation, the findings of increased teeth avulsion frequencies and crown–root fractures, support the thesis that higher axial forces lead to more severe trauma of the teeth and periodontal structures. This explanation can also be used for the significantly increased frequencies of alveolar fractures, which require higher force and trauma to the maxillofacial area. Therefore, the numbers of combined tooth fractures with concomitant periodontal trauma and/or dislocation are only consequent. In addition to that, the increased numbers of pulp capping and rigid splinting (i.e., alveolar fracture) can also be explained by the increased numbers and higher severities of DT. The increased trauma forces that led to DT also seem to be reflected in the increased number of concomitant soft tissue injuries in the IC period.

The maxillary incisors are the most commonly traumatized teeth in the permanent dentition [13]. There were very few significant differences in tooth location between the PC and IC periods. Generally, maxillary incisors are more frequently traumatized due to their anatomical position and structural characteristics [13]. Positioned at the front of the mouth, they are more exposed and protrusive when compared with other teeth, making them the first point of contact in cases of impact. This protruded position, especially when combined with an overjet, makes maxillary incisors particularly vulnerable during falls, collisions, or any direct blow to the face. Additionally, maxillary incisors have relatively thinner enamel and dentin when compared with molars, which are adapted for grinding rather than direct impact. This thinner structure can make the incisors more susceptible to fractures under direct force. The typical activities leading to dental trauma, such as sports, falls, and accidental impacts, are more likely to affect the front teeth, which are not shielded by other teeth or bone structures. Furthermore, the pandemic posed immense challenges for the global healthcare system [21,24]. In addition to vaccination campaigns and education, nationwide lockdowns were implemented with the aim of preventing the rapid spread of the SARS-CoV-2 virus [20,25]. This led to social distancing, fewer sports activities, fewer concerts and partying, and a reduction in road traffic [26,27]. These effects were also noticeable regarding the etiology of DT in the IC period. A significant decrease in sports accidents, road traffic accidents, interpersonal violence, and alcohol-related accidents were observed throughout the IC period. Similar results were found for DT and maxillofacial fractures [18,19,21,23]. At the same time, the present study and recent publications have shown a large increase in falls and accidents within the home [21,23]. The increase in virus-associated syncope is also only consequent, because SARS-CoV infection can cause severe cardiovascular disease and affect hemodynamics [21,28].

The containment of the pandemic also led to a significant increase in home office workplaces, with the result that people spent a large part of their day at home and less time outside, both professionally and privately [29]. The limitation of social interactions and events resulted in a shift in the timing of trauma incidents. This study, along with similar research, observed a notable increase in maxillofacial trauma during daytime hours, accompanied by a simultaneous decline in DT cases at night [18,19,21]. Furthermore, there was a significant increase in DT during the weekdays in the IC period. These changes have also been demonstrated in comparable studies focusing on maxillofacial fractures [18,21].

This study has certain limitations. It is a single-center study with a retrospective design, which limits its statistical power. While the number of participants and DT cases is comparable to other cranio-maxillofacial trauma centers, a more comprehensive analysis would require multicenter studies.

During the COVID-19 pandemic, many private dental practices reduced their patient intake to comply with public health guidelines, prioritizing emergency cases and postponing elective procedures. This operational shift likely led to an increased referral rate to specialized trauma centers for cases requiring immediate attention, such as DT that could not be deferred. Consequently, this influx of cases to trauma centers may have introduced significant bias in the data collected during the pandemic period. The heightened referral rate could inflate the perceived prevalence and severity of DT cases, as trauma centers may have primarily handled more acute and complex cases compared with the pre-pandemic period, when private practices managed a broader range of dental injuries. This referral pattern might have skewed the analysis by overrepresenting severe cases, potentially misrepresenting the overall incidence and nature of DT in the general population. 

In summary, this study represents one of the first investigations into the effects of the COVID-19 pandemic on DT in Germany. The results of the present study confirm that the COVID-19 pandemic had a significant impact on DT. There were significant changes in the type and number of DT (i.e., more crown–root and alveolar fractures) during the IC period, accompanied by changes in the circumstances leading to DT (i.e., more falls/axial trauma, fewer sports accidents, and less interpersonal violence). There was also a significant increase in accidents at home and domestic violence during the COVID-19 pandemic, with a simultaneous decrease in weekend and night-time trauma leading to DT. This suggests that the lifestyle changes induced by lockdowns, including reduced outdoor activities and increased time spent at home, altered the nature and context of DT occurrences. These findings could guide preventative strategies and emergency response protocols in future pandemic scenarios. Furthermore, it may be necessary, especially during pandemic times, to stock certain materials (e.g., treatment of alveolar fractures) in greater quantities and to prepare medical staff for the impacts of a pandemic in the context of dental trauma.

The extended analysis of the effects of the COVID-19 pandemic on DT in future studies may help to predict the effects of a future pandemic on DT and/or maxillofacial trauma and may possibly reduce these effects through establishing targeted preventive measures.

## Figures and Tables

**Table 1:** Baseline characteristics.

Variable	Total (*n* = 250)	PreCovid (02/2019–01/2020) (*n* = 122)	IntraCovid (02/2020–01/2021) (*n* = 128)	*p*-Value
Age (years)	46.21 (±19.27)	46.23 (±19.49)	46.19 (±19.13)	0.986
Gender				0.418
Male	162 (64.8)	76 (62.3)	86 (67.2)	
Female	88 (35.2)	46 (37.7)	42 (32.8)	

Note: Data presented as mean (SD) and/or absolute values (percentage).

**Table 2:** Type of dental trauma.

Variable	Total (*n* = 250)	PreCovid (02/2019–01/2020) (*n* = 122)	IntraCovid (02/2020–01/2021) (*n* = 128)	*p*–Value
Amount of Teeth	2.04 (±1.36)	1.89 (±1.17)	2.19 (± 1.52)	0.079
Enamel + Dentin Fracture (Uncomplicated Crown Fracture)	176 (70.4)	78 (63.9)	98 (76.6)	**0.021**
Combined Tooth Fracture + Dislocation	154 (61.6)	68 (55.7)	86 (67.2)	**0.047**
Crown–Root Fracture	54 (21.6)	10 (8.2)	44 (43.4)	**<0.001**
Complicated Crown Fracture	48 (19.2)	26 (21.3)	22 (17.2)	0.408
Enamel Fracture Only (Uncomplicated Crown Fracture)	26 (10.4)	16 (13.1)	10 (7.8)	0.170
Root Fracture	16 (6.4)	6 (4.9)	10 (7.8)	0.350
Concussion	74 (29.6)	34 (27.9)	40 (31.3)	0.558
Subluxation	66 (26.4)	24 (19.7)	42 (32.8)	**0.018**
Palatinal Luxation	48 (19.2)	30 (24.6)	18 (14.1)	**0.035**
Avulsion	44 (17.6)	16 (13.1)	28 (21.9)	**0.049**
Extrusive Luxation	26 (10.4)	12 (9.8)	14 (10.9)	0.776
Lateral Luxation	34 (13.6)	16 (13.1)	18 (14.1)	0.827
Intrusive Luxation	20 (8.0)	14 (11.5)	6 (4.7)	**0.048**
Alveolar Fracture	18 (7.2)	2 (1.6)	16 (12.5)	**<0.001**

Note: Data presented as mean (SD) and/or absolute values (percentage). Significant *p*-values are presented in bold.

**Table 3:** Location of dental trauma.

Variable	Total (*n* = 250)	PreCovid (02/2019–01/2020) (*n* = 122)	IntraCovid (02/2020–01/2021) (*n* = 128)	*p*-Value
Maxillary Left Molar	6 (2.4)	4 (3.3)	2 (1.6)	0.375
Maxillary Left Premolar	8 (3.2)	4 (3.3)	4 (3.1)	0.945
Maxillary Left Caninus	16 (6.4)	8 (6.6)	8 (6.3)	0.921
Maxillary Incisor	206 (82.4)	98 (80.3)	108 (84.4)	0.401
Maxillary Right Caninus	10 (4.0)	4 (3.3)	6 (4.7)	0.570
Maxillary Right Premolar	2 (0.8)	0 (0)	2 (1.6)	0.166
Maxillary Right Molar	6 (2.4)	2 (1.6)	4 (3.1)	0.443
Mandibular Left Molar	10 (4.0)	2 (1.6)	8 (6.3)	0.063
Mandibular Left Premolar	10 (4.0)	6 (4.9)	4 (3.1)	0.470
Mandibular Left Caninus	6 (2.4)	2 (1.6)	4 (3.1)	0.443
Mandibular Incisor	26 (10.4)	12 (9.8)	14 (10.9)	0.776
Mandibular Right Caninus	6 (2.4)	0 (0)	6 (4.7)	**0.015**
Mandibular Right Premolar	8 (3.2)	2 (1.6)	6 (4.7)	0.171
Mandibular Right Molar	10 (4.0)	4 (3.3)	6 (4.7)	0.570
Combined Mandibular + Maxillary Teeth	16 (6.4)	6 (4.9)	10 (7.8)	0.350
Concomitant Soft Tissue Injury	156 (62.4)	68 (55.7)	88 (68.8)	**0.034**
Concomitant Facial Fracture (excl. Alveolar Fracture)	36 (14.4)	14 (11.5)	22 (17.2)	0.198

Note: Data presented as absolute values (percentage). Significant *p*-values are presented in bold.

**Table 4:** Emergency treatment of dental trauma.

Variable	Total (*n* = 250)	PreCovid (02/2019–01/2020) (*n* = 122)	IntraCovid (02/2020–01/2021) (*n* = 128)	*p*-Value
Composite	208 (83.2)	98 (80.3)	110 (85.9)	0.236
Pulp capping	84 (33.6)	28 (23.0)	56 (43.8)	<0.001
Semirigid Splint (TTS)	150 (60.0)	70 (57.4)	80 (62.5)	0.079
Rigid Splint	22 (8.8)	4 (3.3)	18 (14.1)	**0.003**

Note: Data presented as absolute values and percentage. Significant *p*-values are presented in bold.

**Table 5:** Circumstances of dental trauma.

Variable	Total (*n* = 250)	PreCovid (02/2019–01/2020) (*n* = 122)	IntraCovid (02/2020–01/2021) (*n* = 128)	*p*-Value
Fall	76 (30.4)	16 (13.1)	60 (46.9)	<0.001
Sports accident	62 (24.8)	48 (39.3)	14 (10.9)	<0.001
Epileptic accident	8 (3.2)	4 (3.3)	4 (3.1)	0.945
Road Traffic accident	84 (33.6)	48 (39.3)	36 (28.1)	0.008
Car	12 (4.8)	6 (4.9)	6 (4.7)	0.932
Bicycle	44 (17.6)	26 (21.3)	18 (14.1)	0.132
E-Scooter	24 (9.6)	14 (11.5)	10 (7.8)	0.326
Motorcycle	4 (1.6)	2 (1.6)	2 (1.5)	0.961
Interpersonal violence	42 (16.8)	30 (24.6)	12 (9.4)	0.001
Domestic violence	21 (8.4)	7 (5.8)	14 (10.9)	0.078
Accident at home	79 (31.6)	25 (20.5)	54 (42.2)	<0.001
Alcohol-related	72 (28.8)	60 (49.2)	12 (9.4)	<0.001
Flu/Virus-associated Syncope	24 (9.6)	2 (1.6)	22 (17.2)	<0.001
Month				0.241
January	16 (6.4)	8 (6.6)	8 (6.3)	
February	20 (8.0)	12 (9.8)	8 (6.3)	
March	20 (8.0)	16 (13.1)	4 (3.1)	
April	20 (8.0)	12 (9.8)	8 (6.3)	
May	20 (8.0)	8 (6.6)	12 (9.4)	
June	38 (15.2)	8 (6.6)	30 (23.4)	
July	26 (10.4)	14 (11.5)	12 (9.4)	
August	22 (8.8)	8 (6.6)	14 (10.9)	
September	18 (7.2)	6 (4.9)	12 (9.4)	
October	14 (5.6)	6 (4.9)	8 (6.3)	
November	20 (8.0)	14 (11.5)	6 (4.7)	
December	16 (6.4)	10 (8.2)	6 (4.7)	
Weekday	152 (60.8)	64 (52.5)	88 (68.8)	0.008
Weekend	98 (39.2)	58 (47.5)	40 (31.3)	0.008
Daytime of accident				
Morning	82 (32.8)	14 (11.5)	68 (53.1)	<0.001
Evening	114 (45.6)	62 (50.8)	52 (40.6)	0.106
Night	54 (21.6)	46 (37.7)	8 (6.3)	<0.001
Work-related accident	12 (4.8)	8 (6.6)	4 (3.1)	0.204

Note: Data presented as absolute values and percentage. Morning = 8 a.m.–4 p.m.; evening = 4 p.m.–12 p.m.; night = 12 a.m.–8 a.m. Significant *p*-values are presented in bold.

## Data Availability

Data are contained within the article.
